# Comprehensive Rehabilitation Strategies in Esophageal Cancer: A Case Report of Enhancing Recovery and Quality of Life

**DOI:** 10.7759/cureus.57893

**Published:** 2024-04-09

**Authors:** Rishika Gabada, Vrushali Athawale

**Affiliations:** 1 Oncology Physiotherapy, Ravi Nair Physiotherapy College, Datta Meghe Institute of Higher Education & Research, Wardha, IND

**Keywords:** quality of life, exercise therapy, chemotherapy, rehabilitation, oesophageal cancer

## Abstract

Esophageal cancer is a significant global health burden; it is the seventh most commonly diagnosed cancer and the sixth leading cause of cancer-related deaths globally. It accounts for 3.2% of newly diagnosed malignancies; adenocarcinoma and squamous cell carcinoma are the most prevalent histological subtypes. Clinical presentation often includes dysphagia, odynophagia, weight loss, and persistent heartburn. Diagnosis is confirmed through endoscopy and imaging studies, with treatment typically involving chemotherapy, surgery, and/or radiation therapy. Physiotherapy plays a crucial role in managing pulmonary complications and improving overall cardiopulmonary function in these patients. We present the case of a 70-year-old woman with esophageal cancer, detailing her symptoms, diagnostic assessment, therapeutic interventions, and outcomes, highlighting the importance of a multidisciplinary approach in managing this challenging condition.

## Introduction

Esophageal cancer is among the most frequently occurring cancers in the GI system [1]. Since most patients with esophageal cancer present with advanced, incurable, or metastatic illness, the prognosis can often be dismal [2]. In the world, it ranks seventh in terms of the incidence of cancer diagnoses (3.2% of cases) and sixth in terms of the major cause of fatalities due to cancer (5.3% of mortality rates), according to the World Health Organization's GLOBOCAN 2018 report [3]. A total of 3.2% of all newly diagnosed cancers worldwide are esophageal cancers, placing it third in the gastrointestinal category after stomach (5.7%) and colorectal (10.2%) cancers [4]. With a male-to-female ratio of 2.4:1, esophageal cancer ranks as the fifth most prevalent cancer in men and the sixth most common in women. [5]. Histologically, esophageal cancers are mostly classified as adenocarcinoma (ADCA) or squamous cell carcinoma (SCC) [6]. Barrett’s esophagus is a major factor that contributes to the increasing occurrence of ADCA in the distal esophagus and gastroesophageal junction (GEJ) [7]. Some genetic variants in the epidermal growth factor and other disorders that cause the esophagus to be exposed to more acid are associated with Barrett’s esophageal metaplasia. These ailments include the utilization of drugs that cause relaxation of the lower esophageal sphincter, scleroderma, Zollinger-Ellison syndrome, and specific medical procedures [8]. An increase in the risk of SCCs in the upper esophagus has been associated with HPV infection [9]. Atrophic gastritis, gastrectomy, achalasia, and caustic strictures are other conditions that can heighten the risk of esophageal SCC [10]. Small, polyp-like growths, regions of damaged epithelial cells, and patches usually located in the center of the esophagus are common sites of development for esophageal SCC [11]. The metaplastic epithelium of Barrett’s esophagus is the source of about 60% of ADCAs in the lower esophagus and the GEJ [12]. As cancer spreads and obstructs the esophagus, the primary symptom of both SCC and esophageal ADCA is usually difficulty swallowing solid foods [13]. Typically, the inability to swallow liquids becomes apparent in the later stages of the illness; weight loss and cachexia often occur due to eating difficulties [14]. In individuals without dysplasia, the incidence rate of ADCA is 1.0 cases per 1,000 person-years. However, if low-grade dysplasia is detected during the initial endoscopy, the incidence rate of ADCA increases to 5.1 cases per 1,000 person-years [15]. Physiotherapists play a vital role in enhancing pulmonary complications from radiation or chemotherapy and improving cardiopulmonary function. Exercise plays a multifaceted role in the life of a cancer survivor. It can help mitigate physical and psychosocial side effects, enhance cardiovascular and metabolic health, boost immune function, restore the balance between proinflammatory and anti-inflammatory processes, reduce healthcare costs, and ultimately improve overall quality of life [16].

## Case presentation

A 70-year-old female patient was admitted to the hospital with a constellation of symptoms that had been troubling her for several weeks. She described difficulty and pain while swallowing, persistent heartburn, noticeable weight loss, abdominal discomfort, and substernal pain. Concerned about these symptoms, she sought medical attention to understand their underlying cause. Given the presentation, investigations were done to rule out the diagnosis. An endoscopy was performed, revealing abnormalities in the esophagus. To gather more detailed information, a contrast-enhanced computed tomography (CECT) scan of the neck and thorax was conducted. These investigations confirmed the presence of esophageal cancer, also known as carcinoma of the esophagus. Acknowledging the severity of the condition, the patient underwent chemotherapy as the primary course of treatment. Throughout her treatment plan, she completed a series of eight chemotherapy cycles. A physiotherapy program was initiated, aimed at addressing mobility issues, strengthening exercises, and managing breathing difficulties. This holistic approach to the patient aimed to support her overall general health and enhance her quality of life throughout her treatment journey.

Clinical findings

The patient experienced a range of concerning symptoms, including difficulty swallowing, particularly with solid foods initially, and progressing to liquids. This was accompanied by painful swallowing, described as a burning sensation or sharp pain in the chest or throat. She also noted unintentional weight loss, likely due to the challenges with eating and swallowing. Furthermore, she reported persistent or worsening heartburn, which may be indicative of gastroesophageal reflux disease. She also described chest pain that could be substernal or radiate to the back, neck, jaw, or arms. Additionally, she noticed hoarseness and changes in voice quality. These symptoms caused fatigue and weakness, further impacting her quality of life.

Diagnostic assessment

**Figure 1:**
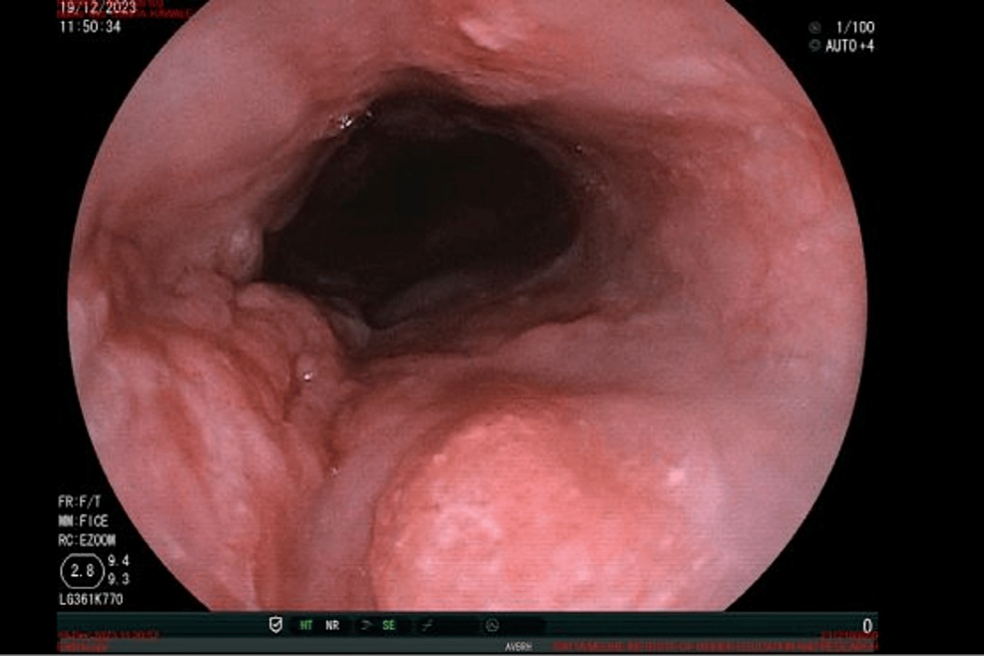
Endoscopy image showing multiple ulcerated lesions in the esophagus

The patient underwent investigations such as endoscopy, revealing multiple discrete, nodular, ulcerated lesions seen over the esophagus starting from 25 cm from the incisor and extending up to 30 cm, sparing the GEJ and not causing luminal narrowing (Figure 1). CECT of the neck and thorax showed long segment thickening of the middle and lower thoracic esophagus. The histopathological report indicated moderately differentiated SCC.

Therapeutic intervention

**Figure 2:**
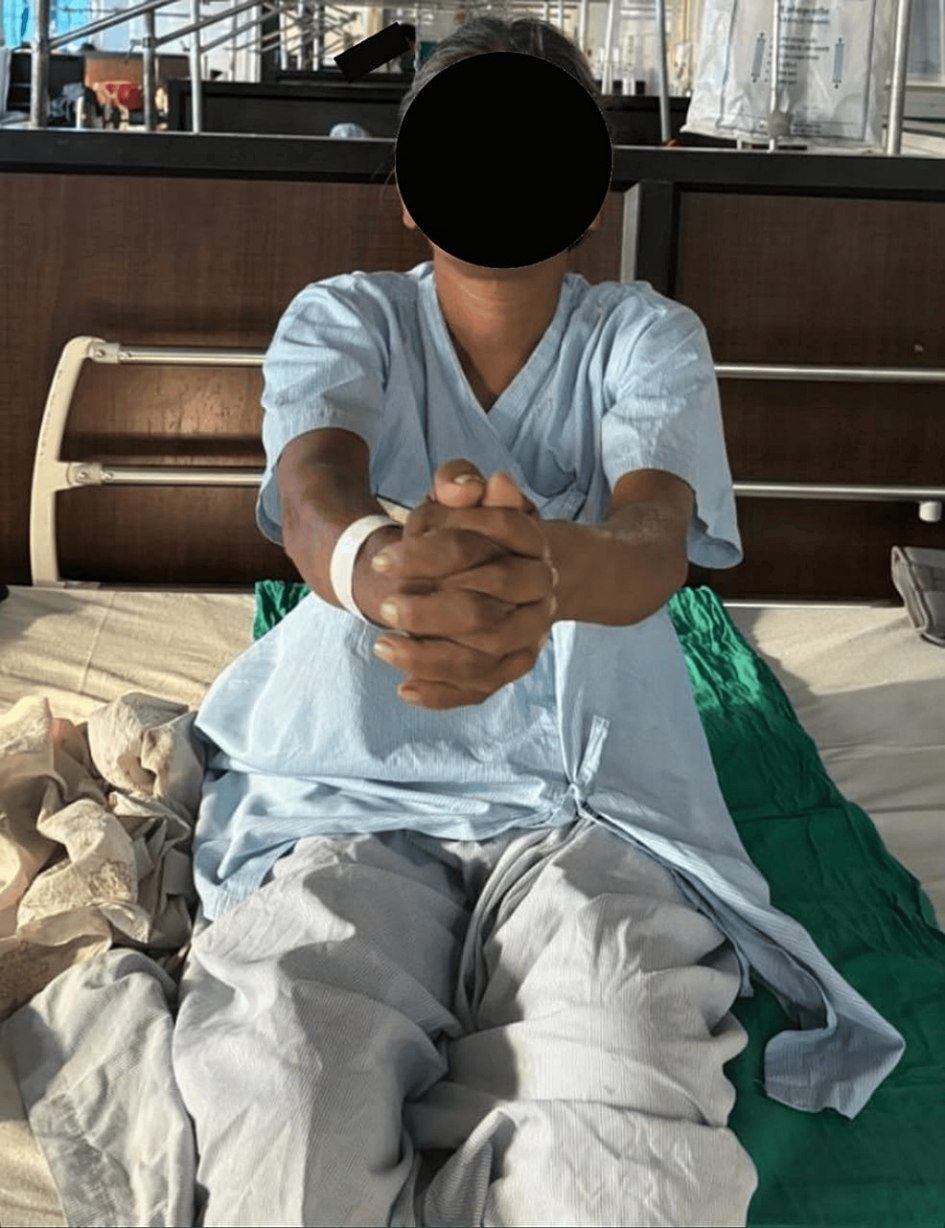
Patient performing thoracic expansion exercises

Table 1 describes the physiotherapy protocol after chemotherapy sessions. Figure 2 shows the rehabilitation course.

**Table 1:** Physiotherapy treatment protocol

Intervention	Description	Duration
Breathing exercises	Diaphragmatic breathing to improve lung function	10 repetitions, 3 times per day
	Deep breathing exercises to expand lung capacity	10 repetitions, 3 times per day
	Thoracic expansion exercises	10 repetitions, 3 times per day
Strengthening exercises	Gentle abdominal strengthening exercises to improve core strength	10 repetitions, 3 times per day
	Upper body strengthening exercises like arm curls	10 repetitions, 3 times per day
	Lower body strengthening exercises like static quads and hams	10 repetitions, 3 times per day
Postural training	Exercises to improve posture and prevent complications	
	Chin tucks: tuck the chin inward and hold for five seconds	10 repetitions, 3 times per day
	Shoulder blade squeeze: squeeze shoulder blades together	10 repetitions, 3 times per day
	Wall angles: stand with back against the wall and raise arms	10 repetitions, 3 times per day
Relaxation techniques	Progressive muscle relaxation	10 repetitions, 3 times per day
Fatigue management	Energy conservation techniques and pacing activities	Integrated into the daily routine

Follow-up and outcomes

Outcome measures post-rehabilitation are summarized in Table 2.

**Table 2:** Outcome measures NPRS, Numerical Pain Rating Scale; MMT, Manual Muscle Testing; WHOQOL, World Health Organization Quality of Life

Outcomes	Pretreatment	Posttreatment
NPRS	7/10	3/10
MMT	3/5	4/5
WHOQOL	45/100	77/100

## Discussion

Esophageal cancer presents significant challenges due to its late diagnosis and limited treatment options. However, advancements in early detection and supportive care are improving outcomes. This case highlights the integral role of physiotherapy in holistic cancer management. Continued collaborative efforts and innovative approaches are essential for further enhancing care and outcomes for patients with esophageal cancer.

In 2017, Guinan et al. conducted a review discussing the role of physiotherapy, encompassing exercise therapy, in the care of individuals with esophageal cancer. The review highlighted that physiotherapy’s traditional focus following esophagectomy has been on immediate postoperative care and the management of postoperative pulmonary complications. While physiotherapy remains a key component of post-esophagectomy care, the review noted a lack of strong evidence supporting specific interventions. It suggested that further research, particularly into early mobilization and alternative postoperative exercise methods, is necessary to better understand their efficacy in improving patient outcomes [17].

In 2018, Bennett et al. conducted a study focusing on the impact of a multidisciplinary rehabilitative intervention involving exercise on individuals who survived esophagogastric cancer. The research highlighted the intervention’s ability to mitigate feelings of isolation commonly experienced by survivors, providing them with essential support in addressing their physical, emotional, and social needs during their recovery process. The study’s results emphasized the significance of tailored rehabilitative programs in meeting the comprehensive needs of esophagogastric cancer survivors. By offering a supportive environment and targeted interventions, these programs can enhance survivors’ quality of life and overall well-being. The findings suggest that integrating exercise and multidisciplinary approaches into survivorship care can play a crucial role in assisting survivors in coping with the physical and emotional challenges of cancer treatment, facilitating a smoother transition to life after cancer [18].

In 2022, Zylstra et al. conducted research, and according to a study, people with esophageal cancer may respond better to chemotherapy if they participate in a planned fitness program before rehabilitation. The study found differences between the intervention and control groups in biochemical and CT-based metrics, which suggested that the intervention group had better body composition, heightened immune function, and decreased inflammation. While the study’s limitations require cautious interpretation, the results underscore the potential benefits of exercise in cancer care [19].

In a 2023 study, Prasad et al. described the Fitness AfteR Oesophagectomy (FARO) study, which aims to determine whether a patient-directed, home-based rehabilitation program can improve quality of life and physical functioning recovery following esophageal cancer surgery [20].

## Conclusions

Esophageal cancer presents a significant challenge, often diagnosed late and with limited treatment options. However, advancements in early detection, treatment, and supportive care are improving outcomes. The case highlights the vital role of physiotherapy in holistic cancer management, addressing physical and emotional needs. Collaborative efforts and innovative approaches are key to further improving care and outcomes for esophageal cancer patients, aiming for better survival rates and quality of life.
